# A comparative study of deep learning‐based knowledge‐based planning methods for 3D dose distribution prediction of head and neck

**DOI:** 10.1002/acm2.14015

**Published:** 2023-05-03

**Authors:** Alexander F. I. Osman, Nissren M. Tamam, Yousif A. M. Yousif

**Affiliations:** ^1^ Department of Medical Physics Al‐Neelain University Khartoum Sudan; ^2^ Department of Physics College of Science Princess Nourah bint Abdulrahman University Riyadh Saudi Arabia; ^3^ Department of Radiation Oncology North West Cancer Centre – Tamworth Hospital Tamworth Australia

**Keywords:** 3D dose prediction, attention neural network, deep learning, head and neck cancer, intensity‐modulated radiation therapy, knowledge‐based planning, radiation therapy, residual neural network, treatment planning

## Abstract

**Purpose:**

In this paper, we compare four novel knowledge‐based planning (KBP) algorithms using deep learning to predict three‐dimensional (3D) dose distributions of head and neck plans using the same patients’ dataset and quantitative assessment metrics.

**Methods:**

A dataset of 340 oropharyngeal cancer patients treated with intensity‐modulated radiation therapy was used in this study, which represents the AAPM OpenKBP – 2020 Grand Challenge dataset. Four 3D convolutional neural network architectures were built. The models were trained on 64% of the data set and validated on 16% for voxel‐wise dose predictions: U‐Net, attention U‐Net, residual U‐Net (Res U‐Net), and attention Res U‐Net. The trained models were then evaluated for their performance on a test data set (20% of the data) by comparing the predicted dose distributions against the ground‐truth using dose statistics and dose‐volume indices.

**Results:**

The four KBP dose prediction models exhibited promising performance with an averaged mean absolute dose error within the body contour <3 Gy on 68 plans in the test set. The average difference in predicting the *D*_99_ index for all targets was 0.92 Gy (*p =* 0.51) for attention Res U‐Net, 0.94 Gy (*p =* 0.40) for Res U‐Net, 2.94 Gy (*p =* 0.09) for attention U‐Net, and 3.51 Gy (*p =* 0.08) for U‐Net. For the OARs, the values for the $D_{max}$ and $D_{mean}$ indices were 2.72 Gy (*p* < 0.01) for attention Res U‐Net, 2.94 Gy (*p* < 0.01) for Res U‐Net, 1.10 Gy (*p* < 0.01) for attention U‐Net, 0.84 Gy (*p* < 0.29) for U‐Net.

**Conclusion:**

All models demonstrated almost comparable performance for voxel‐wise dose prediction. KBP models that employ 3D U‐Net architecture as a base could be deployed for clinical use to improve cancer patient treatment by creating plans with consistent quality and making the radiotherapy workflow more efficient.

## 1 INTRODUCTION

Radiotherapy inverse planning techniques, such as intensity‐modulated radiation therapy (IMRT) and volumetric‐modulated arc therapy (VMAT), can achieve highly conformal dose distribution with decent organ at risk (OAR) sparing results which decrease the risk of adverse events post‐treatment.^1^, ^2^ However, these techniques are technically challenging and involve an iterative back‐and‐forth manner.^3^ Generating a clinically acceptable treatment plan requires domain expertise and may take several hours or even days.^3^, ^4^ Furthermore, the quality of the clinical plan created for the same clinical cases varies substantially across different institutions due to the variation of planners’ experience and institutional practice preferences.^5^, ^6^, ^7^, ^8^ This variability may lead to suboptimal plans that could compromise the final treatment outcome.^9^, ^10^

Data‐driven knowledge‐based planning (KBP) concept has been introduced to improve the treatment planning efficiency and plan consistency/quality.^11^, ^12^ This approach uses knowledge from prior high‐quality clinically acceptable plans to predict various dosimetry features (e.g., dose‐volume metrics, dose‐volume histogram (DVH), or voxel‐wise spatial dose distribution) of a new patient plan. It assumes the existence of a correlation between patient anatomical geometry and delivered dose (achievable plan dose distribution). In other words, the level of achievable OAR dose sparing depends on the proximity of the OAR relative to the planning target volume (PTV). This correlation can be learned by deep learning‐based algorithms. Other sources of knowledge, such as the clinician's experience and treatment trade‐offs, are considered to be embedded in the design of prior clinical plans.

At the early stage of KBP, the concentration was on predicting the dose‐volume metrics to provide prior information to the physician for decision‐making. Subsequently, the DVHs have been utilized to guide the optimization objectives in the treatment planning system. This step has led to minimizing variations among the planners, which resulted in improved plan quality and efficient treatment planning optimization.^12^, ^13^, ^14^, ^15^, ^16^, ^17^ Presently, the focus is on predicting three‐dimensional (3D) dose distributions using deep learning‐based convolutional neural networks.^18^, ^19^, ^20^, ^21^ The 3D predicted dose distribution could then be used as guidance for inverse/objectives optimization planning to generate a deliverable (post‐optimization) radiotherapy plan.^19^, ^22^, ^23^

The head‐and‐neck is one of the most studied treatment sites for KBP dose distribution predictions using deep learning algorithms.^21^, ^24^ It has been recognized as one of the most complex clinical treatment sites due to the proximity and intersection of OARs with multiple targets prescribed to several dose levels. The treatment planning of this site requires a high level of human expertise. Various deep‐learning network architectures have been explored for 2D/3D dose prediction for head and neck treatment. The assessed architectures include residual Net (Res Net),^18^, ^22^, ^25^ hierarchically densely‐connected U‐Net,^26^, ^27^ attention U‐Net,^21^ dilated U‐Net,^28^ cascade U‐Net,^29^ and generative adversarial networks (GANs).^19^, ^20^, ^30^, ^31^ The results reported so far are very promising. However, the performance of these deep‐learning KBP methods for voxel‐wise dose prediction strongly depends on the database used for training. It is challenging to make meaningful comparisons of those proposed KBP models' performance as they were assessed using different data pools and evaluation metrics. The best way for direct comparing the results is to train all models on a same data set then evaluate the trained models on a given testing data set. In this study, we aim to evaluate the performance of various novel KBP methods using 3D deep learning‐based algorithms for head and neck IMRT full 3D dose predictions. The state‐of‐the‐art deep learning 3D convolutional neural network architectures studied here include plain/standard U‐Net, attention U‐Net, residual U‐Net (Res U‐Net), and attention Res U‐Net. This strengthens the current study and makes it more comprehensive compared to our previous work^21^ which included only plain U‐Net and attention U‐Net. The models were evaluated on the same data set using similar assessment metrics. This approach provides direct comparison across the KBP methods and consensus on a scheme of assessment reflecting the critical performance parameters.

## 2 MATERIALS AND METHODS

### 2.1 Patient data and preprocessing

The patients’ dataset used in this study is the Open Knowledge‐Based Planning Grand Challenge (OpenKBP‐Grand Challenge) data.^32^, ^33^ It consisted of 340 oropharyngeal cancer patients treated with IMRT technique. Every patient dataset contained a 3D CT scan, contoured structures of PTVs and OARs delineated by clinicians, and a 3D dose distribution. Each PTV or OAR structure had a unique mask. Therefore, there was no overlap between the PTVs and OARs contoured structures. The clinical intent was to deliver 70 Gy to the gross disease (PTV_70), 63 Gy to intermediate‐risk target volumes (PTV_63), and 56 Gy to elective target volumes (PTV_56) in 35 fractions. Each plan had at least one PTV (a PTV_70). The OAR structures included the brainstem, esophagus, right parotid, left parotid, spinal cord, mandible, and larynx. The plans were generated with the IMRT technique, and the dose delivered was acquired. All data were provided with a size of 128 × 128 × 128 voxels. The CT scans data were acquired with different resolutions with an average voxel size of 3.5 × 3.5 × 2 mm^3^.

To learn sufficient information for accurate dose prediction, we preprocessed the obtained data before being used to train the proposed models for dose predictions. The CT images and contour structures were cropped into smaller sizes of 64 × 64 × 64 voxels. This process helps to reduce the non‐informative background area and to fit the data into the memory for training. All contour masks (PTVs and OARs) were provided as binary data. We cropped the CT data to [0:4095] scale then we normalized the data. We also normalized the dose data by dividing it by the prescription dose (i.e., 70 Gy). It has been reported that intensity normalization helps to speed up the training, and dose distribution normalization improves the predictions when CT and contours are used as input data.^34^ Finally, we structured the input data (CT data and contours) representing a 3D tensor of 64 × 64 × 64 with 12 channels. They are arranged in the channels as follows: (1) CT data, (2) PTV_70 masks, (3) PTV_63 masks, (4) PTV_56 masks, (5) body masks, (6) brainstem masks, (7) left parotid masks, (8) right parotid masks, (9) spinal cord masks, (10) mandible masks, (11) esophagus masks, and (12) larynx masks. The missing PTV or OAR structure data in the original clinical plan data are handled by creating a contour tensor filled with zeros. The total number of plans used in this study were randomly split into 64%/16%/20% as training/validation/testing sets.

### 2.2 Network architectures

U‐Net architecture,^35^ initially designed for biomedical image segmentation, has shown promising performance for dose distribution predictions. Here, we evaluate four variant 3D U‐Net‐based architectures for KBP dose distribution predictions of head and neck. These architectures are, namely: (1) 3D U‐Net, (2) 3D attention‐gated U‐Net, (3) 3D Res U‐Net, and (4) 3D attention‐gated Res U‐Net. In all these architectures, CT and contoured structures data pass through consecutive convolution layers, a bottleneck layer, and several deconvolution layers.

#### 2.2.1 3D U‐Net architecture

**FIGURE 1:**
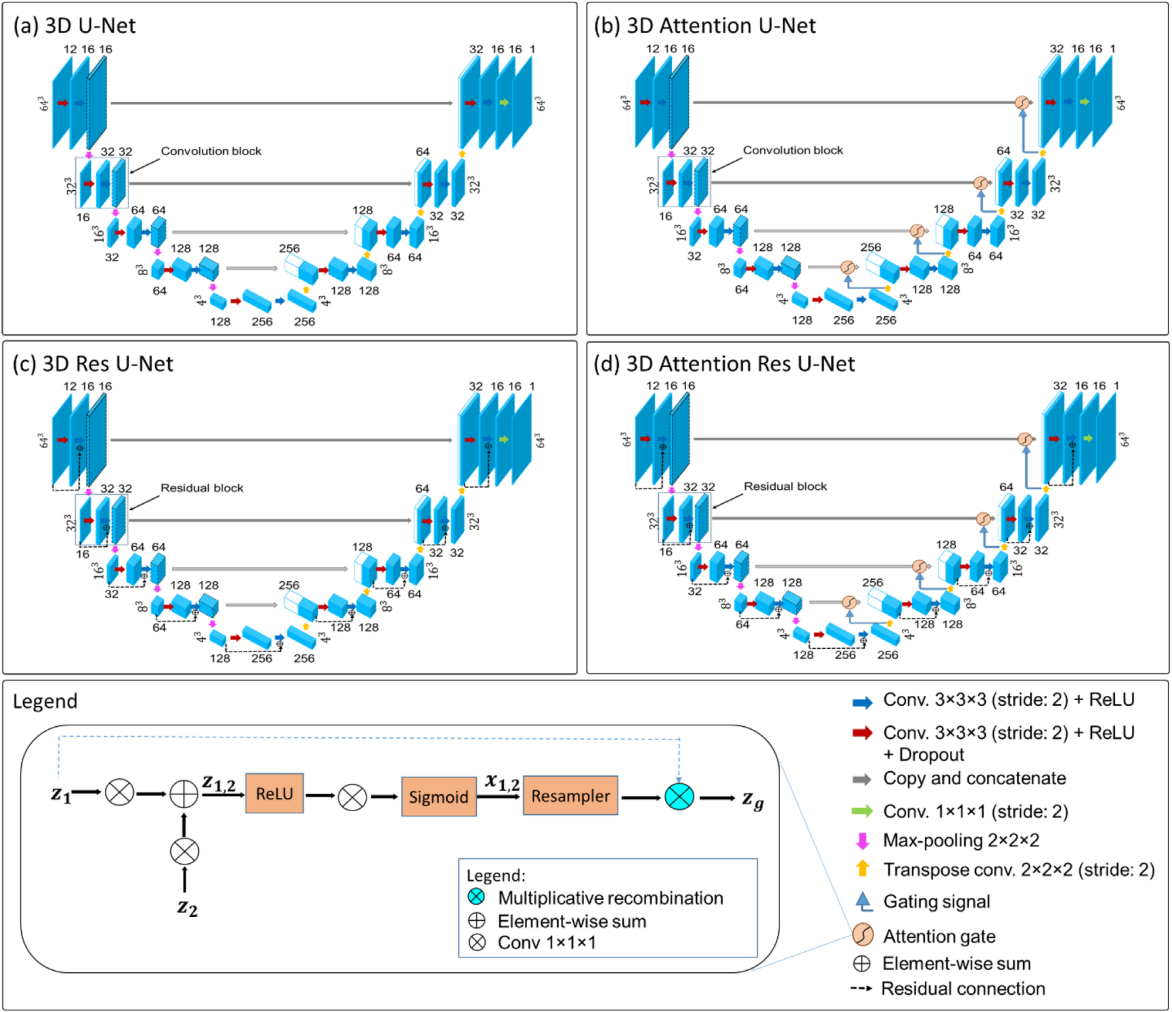
The four 3D U‐Net variant architectures examined for KBP dose predictions in this study. (a) U‐Net, (b) attention‐gated U‐Net, (c) Res U‐Net, and (d) attention‐gated Res U‐Net. Legends that explain all operations are depicted in the figure.

The architecture of the 3D U‐Net we utilized to predict the 3D dose prediction is described in Figure 1(a). It is similar to that used in our previous study.^21^ It is made up of five multi‐scale resolution hierarchical levels.

The encoding part typically contains a convolutional block at each hierarchy level followed by a max‐pooling layer (2 × 2 × 2 kernel size; voxel stride = 2), except at the last one. The convolutional block composes of two 3D convolutional layers (3 × 3 × 3 kernel size; voxel stride = 2), and each convolutional operation is followed by a rectified linear unit (ReLU) layer^36^ as the activation function. The max‐pooling operation employed between every two consecutive hierarchy levels is to successively decrease the size of the feature maps (from 64 × 64 × 64 to 4 × 4 × 4) while the number of derived features simultaneously increases (from 16 to 256) by a factor of 2 at each level. Zero padding is added to the convolution process to maintain the feature size constant. After each convolutional block, the dropout mechanism^37^ is used with an increased increment of 5% (from 10% to 30%) following each hierarchy level to mitigate overfitting and encourage the model to use all of the available filters within the network. We empirically found this incremental scheme of dropout rates yielding the best results for dose predictions.

The decoding part, on the other side, is typically a mirrored version of the decoder with the max‐pooling operation substituted with transposed convolution (2 × 2 × 2 kernel size; voxel stride = 2) successively restore the original dimension of the input data. It also continues to learn non‐linear relationships within the input data. The convolutional block output in the encoder is concatenated with the input to the corresponding one in the decoder. These connections that merge the global abstraction features and spatial features of the same size help recover a clean image.

The final layer in the network is a 3D convolution layer (1 × 1 × 1 kernel size; voxel stride = 2) with a linear activation function. It produces voxel‐wise predictions as the network output (i.e., a single‐channel dose distribution tensor with a size of 64 × 64 × 64 voxels).

#### 2.2.2 3D attention‐gated U‐Net architecture

The architecture of 3D attention U‐Net for voxel‐wise dose prediction is shown in Figure 1(b). It is also typical of what we proposed in our previous study.^21^ In this network, we employed the attention mechanism^38^ on top of the 3D U‐Net architecture (Section 2.2.1). This mechanism empowers the network to suppress irrelevant features and highlight salient features useful for the given task propagating through the architecture. The encoder output at every hierarchy level is concatenated to the corresponding one in the decoder through attention‐gated connections. The output of the preceding hierarchy level in the decoder is used as the gaining signal for the attention‐gated skip connections.

The attention‐gating mechanism utilizes additive self‐attention gates to modulate multi‐scale level feature response propagation throughout each network.^39^ It implements a 3D convolution (1 × 1 × 1 kernel size; voxel stride = 1) to a propagation signal (*z*_1_) and a 3D convolution (1 × 1 × 1 kernel size; voxel stride = 2) to a gating signal (*z*_2_). Signals *z*_1_ and *z*_2_ are added together and the combined activations (*z*_1, 2_) are ReLU activated before being passed through a 1 × 1 × 1 convolutional kernel. The output is sigmoidally activated to form *x*_1, 2_. The final gated output signal ($z_{g}$) is formed by multiplying *z*_1_ by *x*_1, 2_.

#### 2.2.3 3D residual U‐Net architecture

The architecture of the 3D Res U‐Net for 3D dose prediction is shown in Figure 1(c). It is developed by adding residual skip connections^40^ to the 3D U‐Net architecture (Section 2.2.1.1) rather than plain connections. Each convolutional block (stack of similar convolutional layers) in the network is replaced with a residual one. The residual block consists of two successive convolutional layers (3 × 3 × 3 kernel size; voxel stride = 2), with each convolutional operation followed by a ReLU. There is also an identity mapping that joins the input and output in the block.

#### 2.2.4 3D attention‐gated residual U‐Net architecture

The architecture of the 3D attention Res U‐Net for KBP voxel‐wise dose prediction is illustrated in Figure 1(d). It is the same as the attention U‐Net (Section 2.2.2), with all convolutional blocks substituted with residual ones.

### 2.3 Models training

The four KBP models were trained to predict a 64 × 64 × 64 voxel dose tensor for clinical plans. The input to each model is a 12‐channel tensor consisting of CT images and contour data. The output is a single‐channel tensor representing the dose distribution. Adam algorithm that uses gradient‐descent^41^ was used with the learning rate set to 0.001 and momentum set to 0.9. The mean‐squared error cost function was employed to minimize the error between the predicted and ground‐truth voxel‐wise dose distributions during the training as:

(1) $$\mathrm{loss}=\frac{1}{n}\sum_{i\;=\;1}^{n}{\left(y_{i,\;predict}-\;y_{i,\;ground\_truth}\right)}^{2}.$$

where *n* is the total number of voxels and $y_{i}$ is the dose value of a given voxel. It has been widely used as a standard cost function in most deep learning‐based dose prediction studies due to its simplicity, well‐behaved gradient, and convexity. The batch size, representing the number of samples per gradient update, was set to 4. We chose this batch size setting due to our memory and computational power constraints. All models were trained from scratch on a 64% (218 plans) training data set using the He et al.^42^ initialization method. They were trained until every model reached the convergence (180–300 epochs), and the best one that had minimal validation loss was saved. During the training, the models were validated on a 16% (54 plans) validation data set. We applied an early‐stopping validation technique (the patience parameter was set to 20) to monitor the validation loss and terminate the training process early when there is no improvement. This technique helps to avoid overfitting problems and improves the predictions. No post‐processing was performed, except the predicted dose matrix was renormalized back to the original dose scale by multiplying the elements with the prescription dose value (70 Gy).

The total number of trainable parameters for each model was as follows: 5.9 for U‐Net, 6.7 for attention U‐Net, 6.0 for Res U‐Net, and 6.8 million parameters for attention Res U‐Net model. The training was performed on a computer with an Intel Core i5 processor (2.4 GHz) and 8 GB RAM. The training time ranged from 60 to 100 h. Once each model was well‐trained, it took only a few seconds to predict a full 3D dose distribution for a given patient. All models were implemented on Keras API (version 2.6) with Tensorflow (version 2.6) platform as the backend in Python (version 3.7, Python Software Foundation, Wilmington, DE, USA). The code for the four KBP dose prediction models in this study is made publicly available on GitHub respiratory at https://github.com/afiosman/deep‐learning‐KBP‐methods‐for‐rt‐dose‐prediction.

### 2.4 Models evaluation

Once the models were trained and validated, they were applied to predict a full 3D dose distribution on a test set of 68 plans (20% of the data). The predicted and the ground‐truth dose distributions were compared using the same metrics to assess each model's performance for dose predictions. Dose statistics metrics such as voxel‐wise mean absolute error (MAE), mean square error (MSE), and max error (MaxE) between the ground‐truth and predicted doses were calculated within the body contour. Voxel‐wise dose error maps or residuals (predicteddose−ground_truthdose) were plotted to visually inspect the deviations. In addition, dose‐volume indices that are used clinically as a metric for head and neck treatment plan evaluation were calculated to compare the predicted and ground‐truth dose distributions. These indices included the dose to 99% of the volume or the minimum dose received by 99% of the target (*D*_99_) for PTVs, mean dose to a structure ($D_{mean}$) for OARs, and maximum dose to a structure ($D_{max}$) for OARs. DVH curves, representing a histogram in a 2D graphical format relating radiation dose to organ volume, were plotted for all PTVs and OARs. Paired *t*‐test (two‐tail) analysis with a significance level of 0.05 was performed to assess if the differences between the obtained results are statistically significant.

## 3 RESULTS

### 3.1 Dose distribution comparison

**FIGURE 2:**
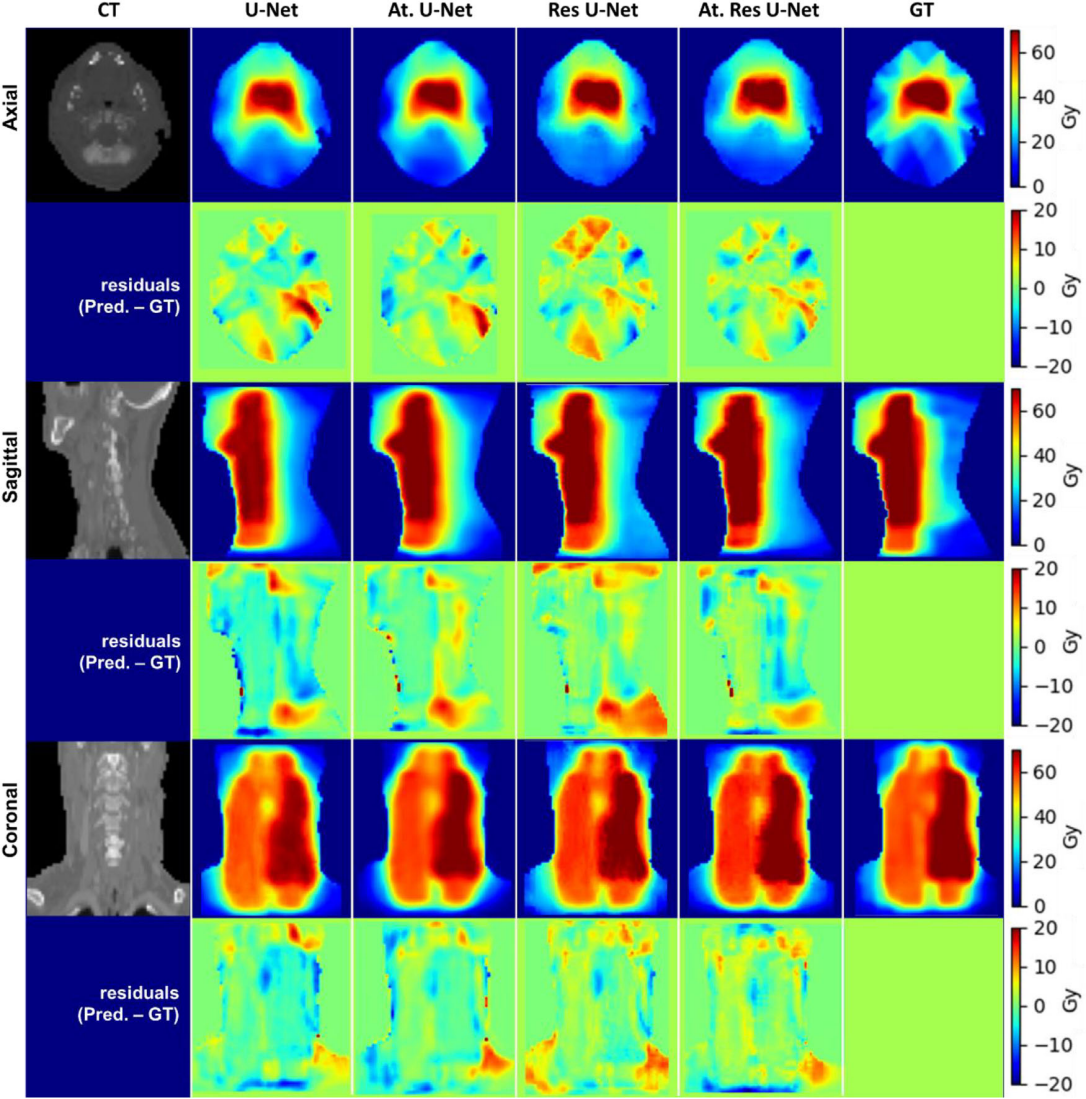
An example of head and neck KBP dose distribution prediction results side‐by‐side with the ground‐truth displayed on different views (axial, coronal, and sagittal) for one patient in the test set. Columns demonstrate the CT image and dose distribution results obtained by 3D U‐Net, 3D attention U‐Net, 3D Res U‐Net, 3D attention Res U‐Net, and the ground‐truth, respectively. Rows indicate different views and the corresponding dose difference maps.

**FIGURE 3:**
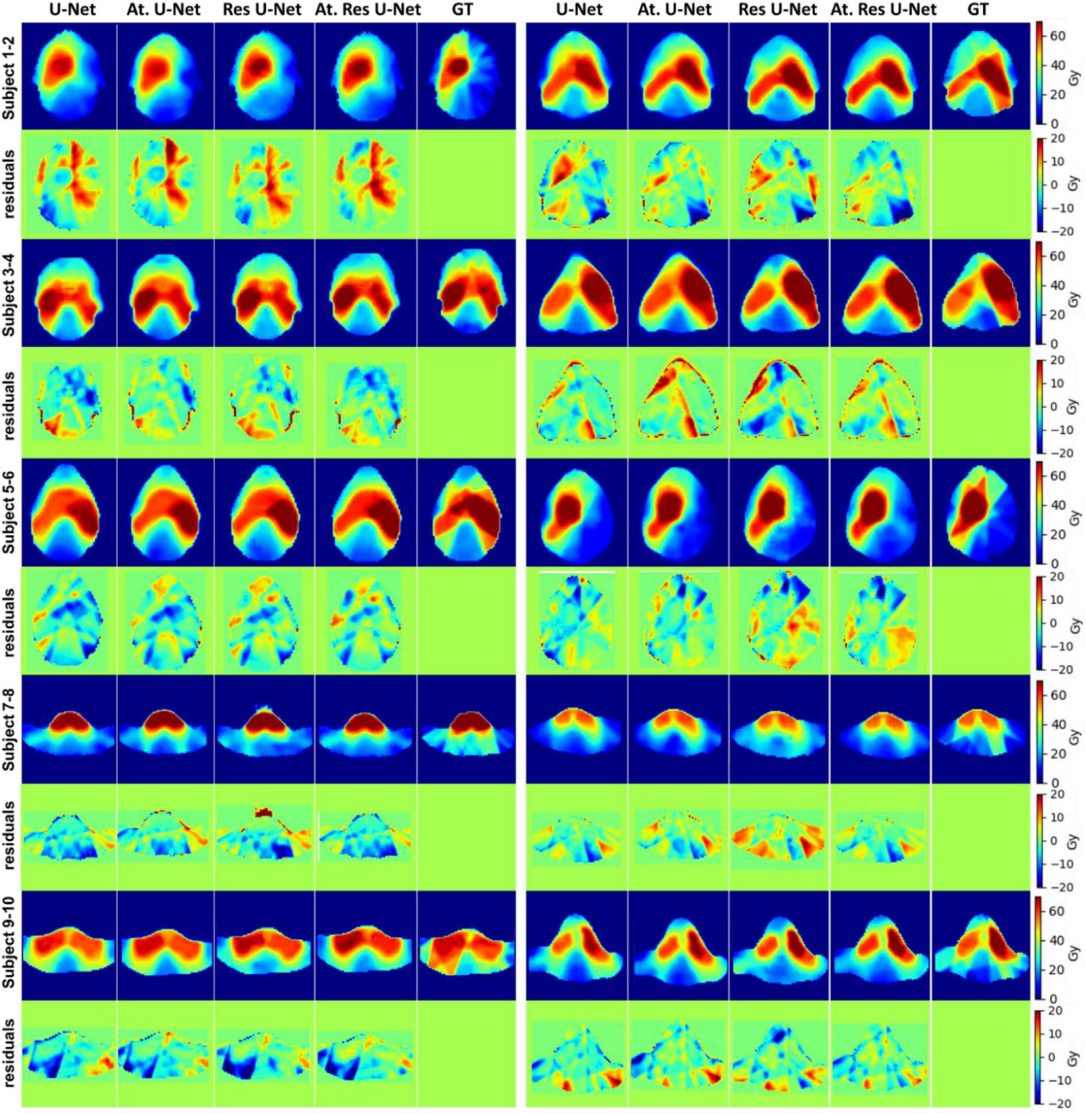
Additional examples of head and neck KBP dose distibution prediction results with the ground‐truth shown on an axial view for 10 patients in the test set. Columns demonstrate the results by 3D U‐Net, 3D attention U‐Net, 3D Res U‐Net, 3D attention Res U‐Net, and the ground‐truth, respectively. Rows indicate different patient examples and the corresponding dose difference maps.

The results of the four KBP dose prediction models that use variant 3D U‐Net architectures are presented in Figure 2 for one patient in the test data set. The predicted dose distributions are shown together with the ground‐truth on three different views (axial, coronal, and sagittal). The residual or voxel‐wise dose error map between the predicted and ground‐truth dose distributions is also shown in the figure for each model. More examples of the prediction results are displayed in Figure 3 on axial view. Both figures, Figure 2 and 3, enable visual inspection of the predicted dose distributions and the locations of the hot/cold spots and dose gradients. Direct comparison among the models shows that the four models provide decent results, and the predicted dose distributions are reasonably similar to the ground‐truth. The figures also show minimal residuals for the four prediction models. There are slight variations from visual inspection of the results to compare the models; however, the four models look to provide similar dose predictions.

### 3.2 Dose statistics comparison

The results of the quantitative evaluation of the predicted dose distributions are presented in Table 1. The MSE, MaxE, and MAE dose were calculated within the body contour and averaged over 68 patients in the test set. The MAE value (from lower to higher) was 2.38 Gy (*p* < 0.01) for attention U‐Net, 2.59 Gy (*p* < 0.01) for Res U‐Net, 2.67 Gy (*p* < 0.01) for attention Res U‐Net, and 2.80 Gy (*p* < 0.01) for U‐Net model. For the MSE metric, its value was 9.52 Gy (*p* < 0.01) for Res U‐Net, 10.29 Gy (*p* < 0.01) for attention U‐Net, 11.07 Gy (*p* < 0.01) for attention Res U‐Net, and 16.48 Gy (*p* < 0.01) for U‐Net. Finally, the MaxE value was 68.36 Gy for attention U‐Net, 71.14 Gy for U‐Net, 71.43 Gy for attention Res U‐Net, and 75.65 Gy for Res U‐Net.

**TABLE 1:** Dose statistics metrics used for evaluating four KBP dose prediction models.

Dose statistics metric	Models			
	U‐Net	Atten. U‐Net	Res U‐Net	Atten. Res U‐Net
MAE (Gy)	2.80 ± 2.62	**2.38 ± 2.15**	2.59 ± 1.68	2.67 ± 1.96
	(*p* < 0.01)	(*p* < 0.01)	(*p* < 0.01)	(*p* < 0.01)
MSE (Gy)	16.48 ± 25.96	10.29 ± 16.98	**9.52 ± 11.53**	11.07 ± 13.55
	(*p* < 0.01)	(*p* < 0.01)	(*p* < 0.01)	(*p* < 0.01)
MaxE (Gy)	71.14	**68.36**	75.65	71.43

The MAE, MSE, and MaxE in the predicted dose distributions were computed within the body contour on the test set. Results are reported as the mean value ± 1 standard deviation. The *p*‐values are also shown.

Bold indicates the best results (the lowest prediction error).

### 3.3 Dose‐volume statistics and DVH comparison

Dose‐volume index provides a clinical perspective assessment of the dose versus volumes (i.e., OARs sparing and target coverage). Dose‐volume indices were computed for every PTV and OAR from the predicted KBP plans with the four algorithms and the ground‐truth plans to provide a clinical measure of prediction quality. Table 2 presents the results of various dose‐volume indices used in the clinic for evaluating the PTVs and OARs of a head and neck plan. The dosimetric indices’ results in the table are reported for the predicted and ground‐truth plans averaged over the 68 patients in the test set. These indices include *D*_99_ for PTVs (PTV_70, PTV_63, and PTV_56) and $D_{mean}$ & $D_{max}$ for OARs (brainstem, left parotid, right parotid, spinal cord, mandible, esophagus, and larynx) evaluation. Table 3 shows the average dose differences in the calculated dosimetric indices between the predicted and the ground‐truth plans (predicted dose—ground‐truth dose). The percentage dose difference values (with respect to the ground‐truth dose) are also presented in the table. The difference in predicting all PTV and OAR indices was 1.63 Gy (*p =* 0.04) for attention Res U‐Net, 1.77 Gy (*p =* 0.02) for Res U‐Net, 0.11 Gy (*p =* 0.98) for attention U‐Net, and 0.46 Gy (*p =* 0.61) for U‐Net.

**TABLE 2:** Dose‐volume indices (PTVs and OARs) for head and neck plan evaluation predicted by each model on the test set (*n* = 68 patients)

	Predicted plans (Gy)				Ground‐
Dose‐volume index	U‐Net	Atten. U‐Net	Res U‐Net	Atten. Res U‐Net	truth plans (Gy)
PTV_70 *D*_99_ (Gy)	62.74 ± 1.63	63.75 ± 2.06	**65.69 ± 1.59**	66.90 ± 2.25	66.99 ± 3.27
	(*p* < 0.01)	(*p* < 0.01)	(*p* < 0.01)	(*p =* 0.80)	
PTV_63 *D*_99_ (Gy)	56.15 ± 3.51	56.55 ± 6.12	**58.71 ± 2.87**	57.80 ± 8.44	61.03 ± 4.62
	(*p* < 0.01)	(*p* < 0.01)	(*p =* 0.01)	(*p =* 0.04)	
PTV_56 *D*_99_ (Gy)	51.91 ± 1.54	**52.20 ± 1.86**	54.09 ± 2.31	53.87 ± 1.78	53.31 ± 3.15
	(*p =* 0.01)	(*p* < 0.01)	(*p =* 0.09)	(*p =* 0.16)	
Brainstem $D_{max}$ (Gy)	36.19 ± 7.59	**34.96 ± 8.26**	36.36 ± 8.92	35.31 ± 7.98	33.35 ± 9.23
	(*p* < 0.01)	(*p* < 0.01)	(*p* < 0.01)	(*p* < 0.01)	
Left parotid $D_{mean}$ (Gy)	33.92 ± 11.56	**32.65 ± 12.77**	37.76 ± 11.69	35.96 ± 12.08	34.01 ± 13.15
	(*p =* 0.85)	(*p* < 0.01)	(*p* < 0.01)	(*p* < 0.01)	
Right parotid $D_{mean}$ (Gy)	34.65 ± 12.54	34.31 ± 13.46	37.98 ± 12.14	**37.03 ± 13.58**	34.27 ± 13.40
	(*p =* 0.28)	(*p =* 0.90)	(*p* < 0.01)	(*p* < 0.01)	
Spinal Cord $D_{max}$ (Gy)	38.48 ± 5.84	37.92 ± 5.41	**37.08 ± 5.43**	39.27 ± 6.36	35.56 ± 5.28
	(*p* < 0.01)	(*p* < 0.01)	(*p* < 0.01)	(*p* < 0.01)	
Esophagus $D_{mean}$ (Gy)	40.23 ± 7.67	41.21 ± 8.26	39.89 ± 7.82	**39.48 ± 7.86**	37.81 ± 6.91
	(*p* < 0.01)	(*p* < 0.01)	(*p* < 0.01)	(*p =* 0.02)	
Larynx $D_{mean}$ (Gy)	46.90 ± 12.49	**49.59 ± 13.10**	50.06 ± 13.02	49.60 ± 13.50	47.32 ± 13.23
	(*p =* 0.46)	(*p* < 0.01)	(*p* < 0.01)	(*p* < 0.01)	
Mandible $D_{max}$ (Gy)	**68.20 ± 4.91**	69.72 ± 4.63	74.13 ± 5.17	75.06 ± 5.45	70.37 ± 5.60
	(*p* < 0.01)	(*p =* 0.08)	(*p* < 0.01)	(*p* < 0.01)	
All $D_{mean}$ (Gy)	38.92 ± 6.01	39.44 ± 7.71	41.42 ± 5.83	**40.52 ± 6.23**	38.35 ± 6.22
	(*p =* 0.43)	(*p =* 0.38)	(*p* < 0.01)	(*p* < 0.01)	
All $D_{max}$ (Gy)	47.62 ± 17.86	47.53 ± 19.27	**49.19 ± 21.60**	49.88 ± 21.90	46.42 ± 20.77
	(*p =* 0.54)	(*p =* 0.34)	(*p =* 0.05)	(*p =* 0.04)	
All *D*_99_ (Gy) or all PTVs (Gy)	56.93 ± 5.46	57.50 ± 5.83	59.50 ± 5.84	59.52 ± 6.69	60.44 ± 6.86
	(*p =* 0.08)	(*p =* 0.09)	(*p =* 0.40)	(*p =* 0.51)	
All OARs (Gy)	42.65 ± 12.08	42.91 ± 13.12	44.75 ± 13.78	**44.53 ± 14.29**	41.81 ± 13.48
	(*p =* 0.29)	(*p =* 0.15)	(*p* < 0.01)	(*p* < 0.01)	
All PTVs and OARs (Gy)	46.94 ± 12.31	47.29 ± 13.12	49.18 ± 13.60	**49.03 ± 14.09**	47.40 ± 14.58
	(*p =* 0.61)	(*p =* 0.98)	(*p =* 0.02)	(*p =* 0.04)	

Results are reported in the form of mean value ± 1 standard deviation. The *p*‐values are also shown.

Bold indicates the best results (the lowest prediction error).

**TABLE 3:** Deviations of each model in predicting the dose‐volume indices (PTVs and OARs) for head and neck plans on the test set (n = 68 patients).

Dose‐volume index	Dosimetric difference (Gy) [predicted—ground‐truth] % difference = [dosimetric difference / ground‐truth]			
	U‐Net	Atten. U‐Net	Res U‐Net	Atten. Res U‐Net
PTV_70 *D*_99_ (Gy)	−4.25 ± 3.01 (6.34%)	−3.24 ± 2.29 (4.84%)	−**1.30 ± 0.92 (1.94%)**	−0.09 ± 0.06 (0.13%)
	(*p* < 0.01)	(*p* < 0.01)	(*p* < 0.01)	(*p =* 0.80)
PTV_63 *D*_99_ (Gy)	−4.88 ± 3.45 (7.99%)	−4.48 ± 3.17 (7.35%)	−**2.14 ± 1.64 (3.80%)**	−3.23 ± 2.28 (5.29%)
	(*p* < 0.01)	(*p* < 0.01)	(*p =* 0.01)	(*p =* 0.04)
PTV_56 *D*_99_ (Gy)	−1.39 ± 0.98 (2.61%)	−**1.11 ± 0.78 (2.08%)**	0.78 ± 0.55 (1.47%)	056 ± 0.40 (1.05%)
	(*p* < 0.01)	(*p* < 0.01)	(*p =* 0.09)	(*p =* 0.16)
Brainstem $D_{max}$ (Gy)	2.85 ± 2.01 (8.54%)	**1.61 ± 1.14 (4.84%)**	3.01 ± 2.13 (9.04%)	1.97 ± 1.39 (5.90%)
	(*p* < 0.01)	(*p* < 0.01)	(*p* < 0.01)	(*p* < 0.01)
Left parotid $D_{mean}$ (Gy)	−0.08 ± 0.06 (0.24%)	−**1.36 ± 0.96 (3.99%)**	3.76 ± 2.66 (11.06%)	1.96 ± 1.38 (5.76%)
	(*p =* 0.85)	(*p* < 0.01)	(*p* < 0.01)	(*p* < 0.01)
Right parotid $D_{mean}$ (Gy)	0.38 ± 0.27 (1.11%)	0.04 ± 0.30 (0.12%)	3.70 ± 2.62 (10.81%)	**2.76 ± 1.95 (8.04%)**
	(*p =* 0.28)	(*p =* 0.90)	(*p* < 0.01)	(*p* < 0.01)
Spinal cord $D_{max}$ (Gy)	2.92 ± 2.07 (8.23%)	2.36 ± 1.67 (6.65%)	**1.53 ± 1.08 (4.29%)**	3.71 ± 2.62 (10.44%)
	(*p* < 0.01)	(*p* < 0.01)	(*p* < 0.01)	(*p* < 0.01)
Esophagus $D_{mean}$ (Gy)	2.42 ± 1.71 (6.40%)	3.41 ± 2.41 (9.01%)	2.08 ± 1.47 (5.05%)	**1.68 ± 1.18 (4.43%)**
	(*p* < 0.01)	(*p* < 0.01)	(*p* < 0.01)	(*p =* 0.02)
Larynx $D_{mean}$ (Gy)	−0.42 ± 0.30 (0.89%)	**2.27 ± 1.60 (4.79%)**	2.74 ± 1.93 (5.78%)	2.28 ± 1.61 (4.83%)
	(*p =* 0.46)	(*p* < 0.01)	(*p* < 0.01)	(*p* < 0.01)
Mandible $D_{max}$ (Gy)	−**2.17 ± 1.53 (3.08%)**	−0.65 ± 0.46 (0.92%)	3.76 ± 2.66 (5.35%)	4.69 ± 3.32 (6.69%)
	(*p* < 0.01)	(*p =* 0.08)	(*p* < 0.01)	(*p* < 0.01)
All $D_{mean}$ (Gy)	0.57 ± 0.41 (1.50%)	1.09 ± 0.77 (2.84%)	3.07 ± 2.17 (8.01%)	**2.17 ± 1.53 (5.65%)**
	(*p =* 0.43)	(*p =* 0.38)	(*p* < 0.01)	(*p* < 0.01)
All $D_{max}$ (Gy)	1.20 ± 0.85 (2.59%)	1.11 ± 0.78 (2.39%)	**2.77 ± 1.96 (5.96%)**	3.46 ± 2.45 (7.45%)
	(*p =* 0.54)	(*p =* 0.34)	(*p =* 0.05)	(*p =* 0.04)
All *D*_99_ (Gy) or all PTVs (Gy)	−3.51 ± 2.48 (5.80%)	−2.94 ± 2.08 (4.87%)	−0.94 ± 0.67 (1.56%)	−0.92 ± 0.65 (1.52%)
	(*p =* 0.08)	(*p =* 0.09)	(*p =* 0.40)	(*p =* 0.51)
All OARs (Gy)	0.84 ± 0.60 (2.02%)	1.10 ± 0.78 (2.63%)	2.94 ± 2.08 (7.03%)	**2.72 ± 1.92 (6.05%)**
	(*p =* 0.29)	(*p =* 0.15)	(*p* < 0.01)	(*p* < 0.01)
All PTVs and OARs (Gy)	−0.46 ± 0.33 (0.97%)	−0.11 ± 0.08 (0.24%)	1.77 ± 1.25 (3.74%)	**1.63 ± 1.15 (3.44%)**
	(*p =* 0.61)	(*p =* 0.98)	(*p =* 0.02)	(*p =* 0.04)

Results are reported in the form of mean value ± 1 standard deviation. The *p*‐values are also shown.

Bold indicates the best results (the lowest prediction error).

**FIGURE 4:**
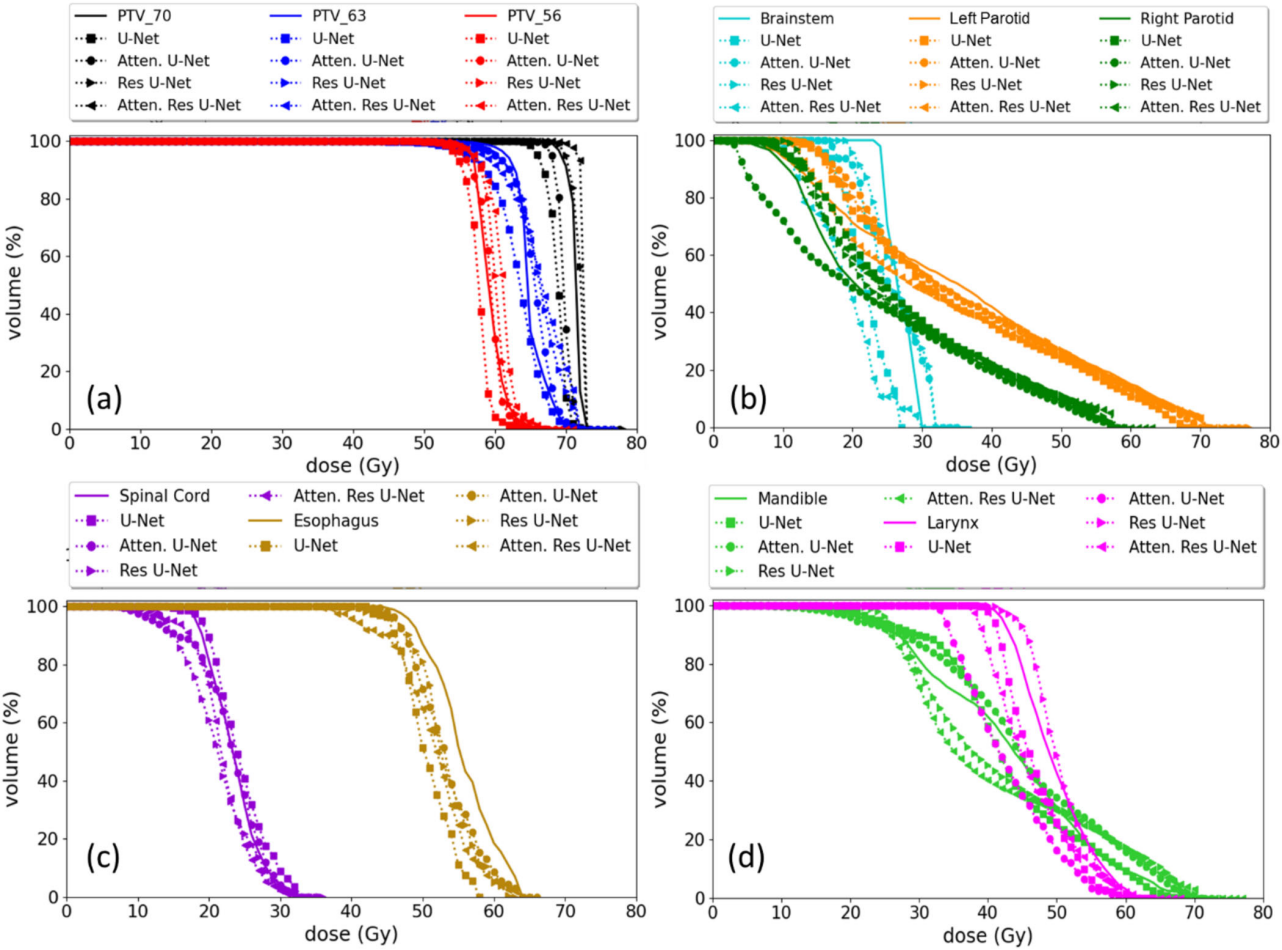
Dose‐volume histogram plots for PTVs and OARs generated using four KBP models compared to ground‐truth for one patent in the test set. DVH curves: ground‐truth (solid line), U‐Net (squares), attention U‐Net (circles), Res U‐Net (right triangles), and attention (left triangles). (a) PTVs curves, (b), (c), and (d) OARs curves.

The DVH curve comparisons (PTVs and OARs) of the four predicted plans and the ground‐truth plan are illustrated in Figure 4 for a representative patient in the test set. All models closely replicated the ground‐truth by predicting DVH curves comparable to the ground‐truth for the PTVs and OARs.

### 3.4 Performance learning curves comparison

**FIGURE 5:**
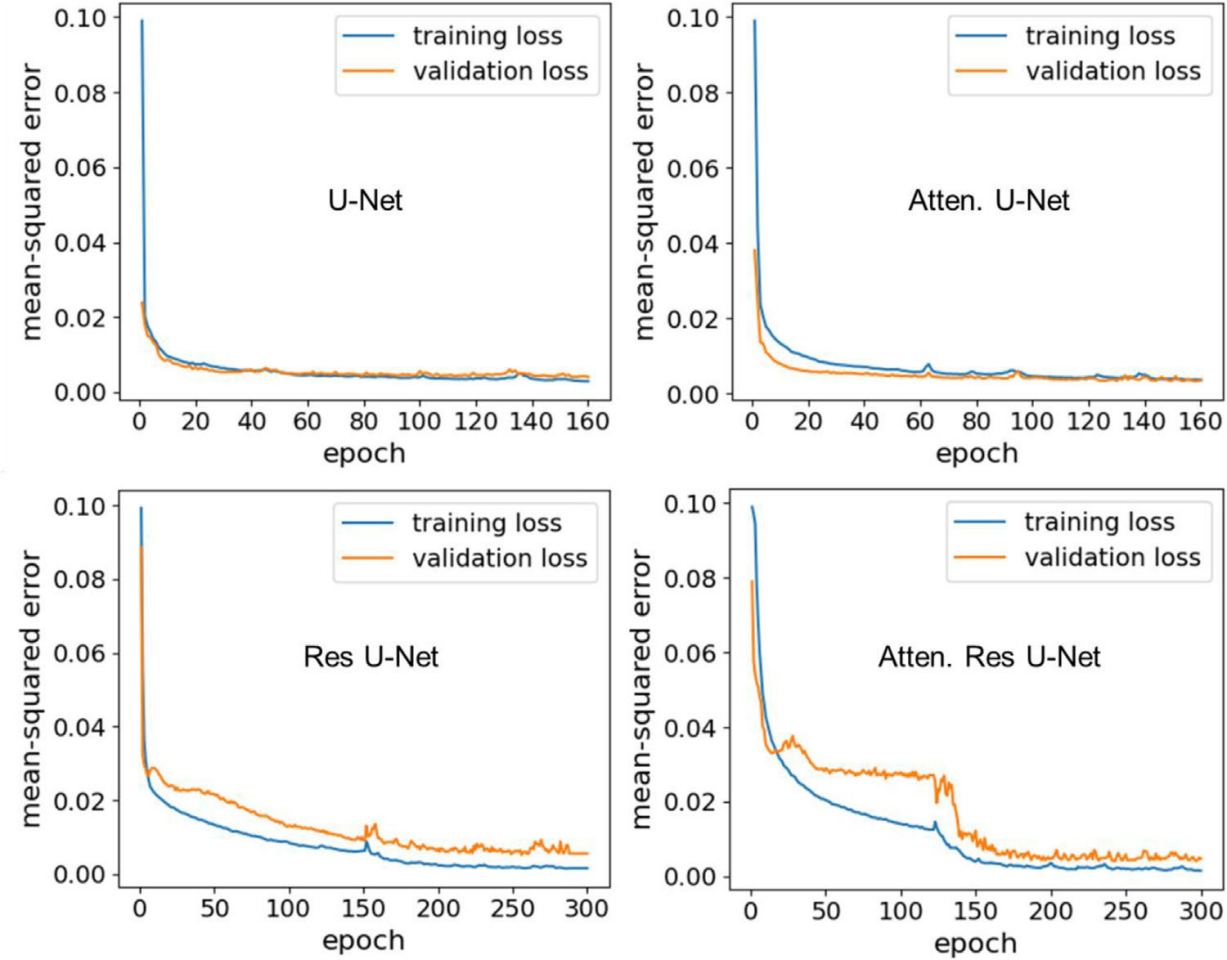
Performance learning curves of the four KBP models.

The learning curve of the model performance provides essential information about the model's generalizability behavior and if the model exhibits underfitting or overfitting performance. Figure 5 shows the performance learning curves of the four dose prediction models. All models demonstrated a good fit for the dose prediction task. The curves for U‐Net and attention U‐Net models showed perfect overlap of the training and validation curves. On the other hand, Res U‐Net and attention Res U‐Net models exhibited minor overfitting performance, indicated by existence of a gap between the training and validation curves. The learning curve of attention Res U‐Net model indicates a sudden change after epoch 120. This is because we trained the model in multiple sessions (i.e., first we trained the model for 120 epochs in session #1, then for another 120 epochs in session #2, and finally for 60 epochs in session #3). Training the model for 300 epochs in a single session is very expensive on our computational resources. All models were trained sufficiently with the given data size until reaching the convergence region. The training loss is always lower than the validation loss. Moreover, the gap between them is minimal, demonstrating good generalizability of the models in dose prediction on new data sets with a minimum margin of error.

## 4 DISCUSSION

It is challenging to make direct comparisons among the existing KBP dose prediction methods since the previous studies were carried out using different patient databases and treatment modalities or protocols. This study aimed to evaluate the performance of four deep‐learning KBP dose prediction methods for head and neck cancer on the same test set using different evaluation criteria. This approach allows a meaningful comparison across the competitive KBP models and provides valuable insight into accuracy. The KBP evaluated methods in this study use the 3D U‐Net architecture as a base, and they include plain/standard 3D U‐Net, 3D attention U‐Net, 3D Res U‐Net, and 3D attention Res U‐Net. All methods use CT and contour data to predict voxel‐wise dose distribution for the head and neck. The networks were constructed to use 3D operations to handle the volumetric data. Having a 3D deep learning architecture enables the model to account for the correlations between the adjacent 2D slices and allows the model to capture additional spatial information. The four models have typical dose prediction pipelines. The main difference is in the network architecture.

The evaluation of predicted distributions showed comparable performance of the four models as they produced dose distributions almost similar to the ground‐truth (Figures 2 and 3). Visual inspection of the predicted dose distributions showed almost comparable performance of all models. From the Figures 2 and 3, we can observe that all models tended to predict higher and smoother (e.g., less dose gradient of IMRT) doses outside the PTV compared to the ground‐truth dose distributions. This is could be due to using coarser resolution (e.g., voxel size 7 × 7 × 4 mm^3^) data to train the models than the original one (e.g., voxel size 3.5 × 3.5 × 2 mm^3^). One possible way to solve this problem could be reducing the voxel size of the input data (using finer resolution); however, this will require more memory and computational powers. The quantitative dose statistics comparison of the four KBP models (Table 1) showed that they achieved an overall low dose prediction errors. The results showed that there are huge uncertainties associated with the calculated dose error metrics (e.g., the MAE and MSE metrics). The differences among the computed metrics were within the one standard deviation which make it difficult to identify the best performance model among the assessed ones. The computed MaxE metric values of the four models were very high (i.e., close to the prescription dose which is 70 Gy); however, this could be just one voxel located adjacent to inner/outer boundaries of the PTV. Overall, the four KBP models demonstrated capability in predicting voxel‐wise doses within 4% MAE within the body contour (relative to the prescription dose). Therefore, they have the potential for clinical deployment.

The KBP models were also quantitatively assessed considering the dose‐volume indices clinically used for evaluating the head and neck plans (Tables 2 and 3). Here also it is hard to determine which model is performing the best from the computed DVH metrics, almost all values are within one standard deviation. The performance of the four KBP models assessed in this study revealed their capabilities in predicting the dose‐volume indices for all PTVs and OARs structures within 3.5%. Clinically acceptable plans typically cannot satisfy all clinical criteria simultaneously because of the proximity of the PTVs to the OARs and the complexity of the head‐and‐neck site. In this study, therefore, we did not assess the predicted plans for this metric.

It is challenging to compare the performance of the evaluated KBP models in this study with the competing ones reported in the literature. The main reason is that those models were developed and assessed using different data sets. However, we made a rough comparison with the top‐three methods in the OpenKBP‐2020 AAPM Grand Challenge competition, which is the most similar to this study. The comparison results showed that the performance of the U‐Net (MAE = 2.80 Gy), attention U‐Net (MAE = 2.38 Gy), Res U‐Net (MAE = 2.59 Gy), and attention Res U‐Net (MAE = 2.67 Gy) was competitive to Liu et al.^29^ method using Cascade 3D U‐Net (MAE = 2.31 Gy), Gronberg et al.^28^ method using 3D dense‐dilated U‐Net (MAE = 2.56 Gy), and Zimmermann et al.^31^ method using GAN (MAE = 2.62 Gy). Moreover, the performance of the KBP models in this study (MAE = 4%, relative to the prescription dose) was superior to Chen et al.^18^ method using Res‐Net‐101 (MAE = 5.3%, relative to the prescription dose).

Comparison of different methods using the same patients’ data pool, treatment planning settings, and evaluation metrics provides valuable insight into robustness, potential applicability, and expected quantification errors in a clinical setting. Once KBP models have been sufficiently trained, the 3D dose prediction for a new patient would take just 6 s. As a result, KBP models have an appealing choice for clinical application. This advantage would be particularly crucial for adaptive radiotherapy planning, which involves the creation of new treatment plans during radiotherapy fractions. The models can also be re‐trained over time by adding new samples to the training dataset to boost their robustness without affecting computational cost. The KBP models could serve as a tool for quality assurance of the clinical treatment plan. Therefore, the planners can know in advance the feasibility of improving the dose distributions of the treatment plan. The physicians can also immediately view 3D dose distributions to adjust the dose constraint requirements for OARs. Meanwhile, the planners could take advantage of these OARs DVHs from dose distributions to define optimization objective function that may improve the quality and consistency of treatment plans and reduce planning time. Post‐optimization could be performed to the predicted 3D dose distributions to generate a deliverable plan.

One of the limitations of this study is that the dataset size used for developing and testing the KBP models in this study is not very large. Having a substantially larger data set would further improve the performance of these models as well as tighten the generalization gap (the gap between the training and validation loss). In addition, the dataset covered only the IMRT delivery technique and omitted VMAT for comprehensive evaluation of the models for head and neck radiotherapy treatment. Furthermore, the KBP models were trained for dose predictions using plans of fixed beam configuration. In routine clinical practices, choosing the beam orientations could broadly differ based on the institution's protocol, the planner's perspective, and the patient condition. Lastly, this study performed a single training and evaluation of the models on the dataset rather than implementing a cross‐validation, that is, train, validate and test all architectures on several different folds of the dataset. This may weaken the conclusion of this study of being fully justified.

## 5 CONCLUSION

Four KBP methods that use 3D U‐Net as a base of architecture were evaluated in this study on a unified data set for voxel‐wise dose predictions of head and neck plans using various assessment metrics. All models demonstrated promising performance and comparable results for accurate 3D dose distribution predictions achieving less than 4% mean absolute dose error. The findings of this study show the potential of the assessed four KBP dose prediction models for clinical application in the automated treatment planning pipeline with tolerable error. Consequently, it would lead to a more efficient clinical operational paradigm that reduces plan generation time and speed up the radiotherapy workflow.

## AUTHOR CONTRIBUTIONS

Alexander F. I. Osman contributed to the conception and design of the study, methodology, technical aspects, drafted the manuscript, and revised it for important intellectual content. Nissren M. Tamam and Yousif A. M. Yousif contributed to writing some sections of the manuscript and revised it for important intellectual content. All authors contributed to manuscript revision and approved the submitted version.

## CONFLICT OF INTEREST STATEMENT

The authors have no conflict of interest to disclose.

## Data Availability

The datasets can be found in the OpenKBP – 2020 AAPM Grand Challenge repository at https://competitions.codalab.org/competitions/23428. The code for the model developed in this study is made available as an open‐source package on GitHub respiratory at https://github.com/afiosman/deep‐learning‐KBP‐methods‐for‐rt‐dose‐prediction.
